# A Thermo–TDR Sensor for Simultaneous Measurement of Unfrozen Water Content and Thermal Conductivity of Frozen Soil

**DOI:** 10.3390/s25072155

**Published:** 2025-03-28

**Authors:** Panting Liu, Simao Fan, Qingyi Mu, Qifan Zhang, Linlin Tang, Jine Liu, Fuqing Cui, Zhiyun Liu, Xuna Wang

**Affiliations:** College of Geology Engineering and Geomatics, Chang’an University, Xi’an 710054, China; 2022126128@chd.edu.cn (P.L.); 2023126128@chd.edu.cn (S.F.); qingyimu@chd.edu.cn (Q.M.); 2024226137@chd.edu.cn (Q.Z.); tangll@chd.edu.cn (L.T.); 2020026010@chd.edu.cn (J.L.); dcdgx33@chd.edu.cn (Z.L.); wangxuna0607@163.com (X.W.)

**Keywords:** multi-sensor fusion, thermo-TDR probe, frozen soil, unfrozen water content, thermal conductivity

## Abstract

Due to increasing human engineering activities in cold regions, the precise measurement of frozen soil’s physical property parameters has become particularly important. Traditional measurements of thermal conductivity and unfrozen water content of frozen soil are usually tested separately, leading to errors in accurately understanding the dynamic variation law of permafrost’s hydrothermal parameters in the near-phase transition zone. To address this, a multi-sensor fusion technology–thermo time domain reflectometry (thermo-TDR) sensor was designed and optimized for measuring the unfrozen water content and thermal conductivity of frozen soil. Three-dimensional thermal and electromagnetic numerical models were developed to analyze and validate the design parameters of the proposed sensor. Furthermore, a corresponding validation experiment was carried out to confirm the usability and accuracy of the designed sensor. The results show that (1) under the optimized probe parameters, the deviation between the theoretical thermal conductivity and the numerical preset value is 2.94%, verifying the accuracy of the sensor in thermal physical testing. (2) With a 10 mm probe spacing design, the test area of the thermo-TDR significantly increased, and the skin effect coefficient reached 25.54%, satisfying the electromagnetic design requirements of the TDR method. (3) The designed thermo-TDR sensor realizes the simultaneous measurement of unfrozen water and thermal conductivity of frozen soil, and the experimental results present a good consistency with that of the nuclear magnetic resonance (NMR) and transient planar heat source methods. (4) Additionally, due to the drastic changes in the soil’s physical properties due to the probe’s heating process, testing errors of the thermo-TDR sensor will significantly increase in the near-phase transition range, especially in the range of −2~−1 °C.

## 1. Introduction

Multi-sensor fusion technology has become a promising development trend in the field of sensor research and manufacture [1]. This trend is mainly due to the fact that in complex and volatile natural environments, a single sensor usually cannot meet the needs of high-precision and multi-dimensional data [2,3]. Therefore, multi-sensor fusion can help overcome these limitations and significantly improve the accuracy, real-time, and reliability of data acquisition [4,5]. The application of multi-sensor fusion technology in permafrost research is of critical significance, especially for the accurate measurement of the physical parameters of frozen soil [6]. Traditional measurement techniques usually rely on a single sensor for multiple separate data acquisition [7], which is often susceptible to different degrees of limitations, such as measurement error, time delay, etc., and cannot achieve continuous measurement of different physical property parameters at the same time and the same volume of soil. In particular, the measurement of unfrozen water content and thermal conductivity in permafrost remains a major challenge because unfrozen water content varies drastically near the freezing point [8,9], and multiple measurements by a single sensor are difficult to capture real-time variations due to the time lag and spatial heterogeneity, often leading to measurement errors, and face trade-offs between cost, resolution, and field applicability [10,11,12,13]. Therefore, there is an urgent need for a multi-sensor fusion technique to overcome the effects of spatial and temporal soil variations on the measurements and, thus, improve the accuracy of the measurement of the physical parameters of frozen soil.

As a multi-sensor fusion technology, the thermo-TDR sensor can measure the soil’s thermal properties and water content simultaneously, which fundamentally avoids the errors caused by sample differences of asynchronous testing. The researchers conducted a series of studies on geometric design parameters to improve the measurement accuracy of the temperature–TDR sensor. Considering that the soil’s thermal properties were mainly derived by the infinite line source (ILS) model, Knight [14] recommended that the ratio of probe spacing-to-diameter should not exceed 10 mm, and the ratio should be minimized as long as there is no significant compaction or localized disturbance. Blackwell [15] noted that to restrict the axial heat flux error to within 1%, the L/d ratio should be greater than 25. Wen et al. [16] extended the probe length to 60 mm and arranged temperature sensors at multiple locations on the probe, allowing in situ correction to measure spacing changes caused by probe deflection. Wei et al. [17] designed a larger thermo-TDR probe with a length of 70 mm and probe spacing of 10 mm and experimentally verified its better measurement accuracy for testing moisture content and dry density. In addition, to minimize interference during insertion, researchers also enhanced the design of the probe. Liu et al. [18] added a pointed tip to the sensor and increased the probe diameter to 2 mm and spacing to 8 mm. The results exhibited that the pointed tip enhanced the convenience of sensor insertion, but the short probe length still limited its measurement precision. Yu et al. [19] proposed a similar probe with a pointed design, which showed a better performance for dense soil samples.

Meanwhile, the thermo-TDR sensor has also been adopted in the testing of permafrost’s physical parameters [20]. For example, He et al. [21] analyzed the main design factors of the thermo-TDR sensor and discussed its prospects and limitations for permafrost measurement, which provided a basis for subsequent research. Topp et al. [22] investigated the effect of sensor geometry on test results and noted that a 50 mm probe length design resulted in significant moisture content measurement errors. Tian et al. [23] proposed a thermo-TDR sensor design that significantly improved the measurement accuracy of the ice content of frozen soil, especially approaching the freezing temperature point. Yuki et al. [24] designed a sensor with a length of 77 mm and proposed a test method to measure ice content at lower temperatures and experimentally confirmed that the thermal Fourier transform temperature sensor can measure ice content over the entire temperature range.

Although there have been some exploratory investigations on the measurement of frozen soil’s physical properties, the thermo-TDR sensor still faces some technical challenges, such as underrepresentation due to a small sampling region and considerable test error in the near-phase transition temperature range. Additionally, it should be noted that in existing thermo-TDR studies, there are few research works on simultaneous measurement of the hydrothermal parameters of frozen soil. Therefore, in this work, a thermo-TDR sensor was designed and optimized for measuring the unfrozen water content and thermal conductivity of frozen soil. The heat transfer and electromagnetic numerical models of the thermo-TDR sensor were developed to analyze the proper probe spacing and energy distribution characteristics. Furthermore, an experimental system of the thermo-TDR sensor was built, and validation experiments of NMR and transient planar heat source methods were carried out to confirm the usability and accuracy of the designed sensor.

## 2. Methods and Materials

### 2.1. Design of Thermo-TDR Sensor

**Figure 1:**
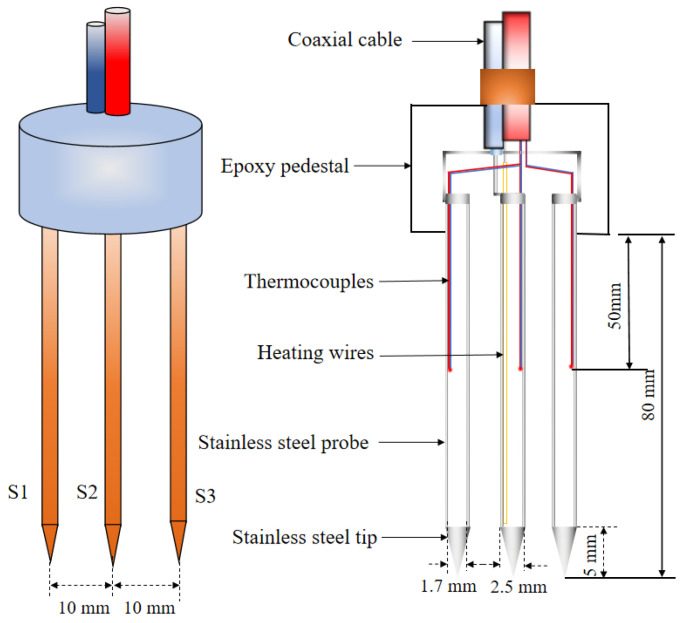
Schematic diagram of thermo-TDR sensor design.

The schematic diagram of the thermo-TDR sensor design is shown in Figure 1. The sensor was composed of a coaxial cable, an epoxy base, and three stainless steel probes. The round base was made of epoxy resin (TH-896W-30, Yanhua Adhesives Co., Xi’an, China), which was cured in prefabricated molds. Tubular stainless steel probes (S1, S2, and S3) were arranged at equal intervals in the center of the base and integrally molded with coaxial cable (SYV75-3-1, Shaanxi North Cable Wire & Cable Co., Xi’an, China). The length of the probes was designed as 80 mm, with inner and outer diameters of 1.7 mm and 2.5 mm, respectively. A T-type thermocouple was embedded in the middle of the probe, with a wire diameter of 0.08 mm and an accuracy of ±0.1 °C. The center probe (S2) was inserted into an 80 mm length electric heating wire, which was made of enameled nickel–chromium resistance wire, and paperclip-folded twice in the probe to reach a resistance of 145 Ω/m. Furthermore, based on the thermal and electromagnetic numerical calculation results (see Section 3.1 for details), the center distance between the probes was determined as 10 mm, which could expand the testing range of the soil sample and ensure the representativeness of the measurement results.

**Figure 2:**
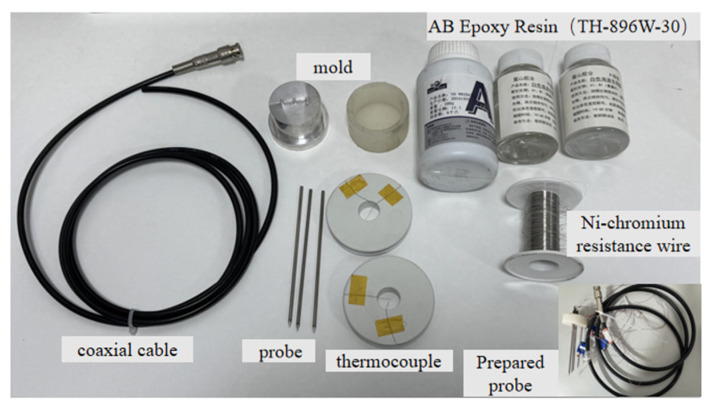
Manufacture components of thermo-TDR sensor.

In the manufacturing process of the sensor, as shown in Figure 2, the stainless steel probe was made of a seamless steel tube welded with a conical head. The built-in thermocouples and heating wires of the probes were coated with a high thermal conductivity epoxy mixture layer to ensure complete electrical insulation with the stainless steel casing. After placing the thermocouples and heating wire into the probe, a high thermal conductivity epoxy resin, which remained in a liquid state at room temperature, was filled into the tube to secure the position of the above components after curing. Additionally, in order to guarantee the installation accuracy of the probe, a silicone mold for the epoxy resin base and a plastic mold for the probe hole position were prefabricated. A coaxial cable with an impedance of 75 Ω was connected to the corresponding probes by welding, allowing electromagnetic waves to propagate through the cable and tubes to achieve TDR functionality. Finally, the epoxy was poured into the mold, and after 48 h of curing, the various parts of the probe were held in place.

The thermal properties test of the thermo-TDR sensor was determined by the theory of ILS [25], which was derived using the test values of the temperature curve of *S*_2_ and *S*_3_*/S*_1_ probes. Based on the ILS theoretical model, the temperature rise at a distance *r* from the *S*_2_ probe (origin point) can be calculated as [26,27]:

(1) $$\Delta T(r,t)=\left\{\begin{array}{ll} -\frac{Q}{4\pi \kappa }Ei\left(-\frac{r^{2}}{4\kappa t}\right) & \left(0<t\leq t_{0}\right)\\ \frac{Q}{4\pi \kappa }\left[Ei\left(\frac{-r^{2}}{4\kappa \left(t-t_{0}\right)}\right)-Ei\left(\frac{-r^{2}}{4\kappa t}\right)\right] & \;(t>t_{0}) \end{array}\right.,$$

where *t* is the current time; *t_0_* is the total heating time of the *S*_2_ probe; Δ*T*(*r*,*t*) indicates the temperature rise at a distance *r*; *κ* is the thermal diffusivity, which is the ratio of thermal conductivity-to-volumetric heat capacity; *Q* is the electrically heated power per unit length of the *S*_2_ probe; *Ei(−x)* is the exponential integral, which can be evaluated using the formula proposed by Abramowitz and Stegun [28].

Employing the temperature curve of the *S*_3_ or *S*_1_ probes, the corresponding time for the maximum temperature rise, *t_m_*, can obtained. The volumetric heat capacity (*C*) and thermal diffusivity (*κ*) can be calculated as:

(2) $$\kappa =\frac{r^{2}}{4}\left[\frac{1/\left(t_{m}-t_{0}\right)-1/t_{m}}{Int_{m}-In\left(t_{m}-t_{0}\right)}\right],$$

(3) $$C=\frac{q}{4\pi \kappa \Delta T_{m}}\left\{Ei\left[-\frac{r^{2}}{4\kappa (t_{m}-t_{0})}\right]-Ei\left[-\frac{r^{2}}{4\kappa t_{m}}\right]\right\},$$

Then, the thermal conductivity can be obtained as:

(4) $$\lambda =\frac{q}{4\pi T_{m}}\left[Ei\left(\frac{-In\left[t_{m}/\left(t_{m}-t_{0}\right)\right]}{t_{0}/t_{m}}\right)-Ei\left(\frac{-In\left[t_{m}/\left(t_{m}-t_{0}\right)\right]}{t_{0}/\left(t_{m}-t_{0}\right)}\right)\right],$$

Regarding the calculation of unfrozen water content, the empirical formula proposed by Siddigui et al. [29] was employed:

(5) $$\sqrt{K_{a}}\frac{\rho_{w}}{\rho_{d}}=a+bw,$$

where *K_a_* is the apparent dielectric constant of the soil sample; *w* is the water content, *ρ_w_* is the density of water; *ρ_d_* is the dry density of the soil; *a*, *b* is the calibration constant of the soil, respectively.

### 2.2. Heat Transfer Simulation to Determine the Probe Parameters

In order to determine the optimal probe spacing, we compared the ILS theoretical solution of temperature rise and thermal conductivity with the numerical calculation results. Different probe spacing conditions were analyzed, i.e., 6 to 12 mm. The theoretical solutions were determined based on the calculation principles of the sensor as described in Section 2.1, and the numerical results were obtained from the transient heat transfer simulation of a heating probe in a semi-infinite space.

**Figure 3:**
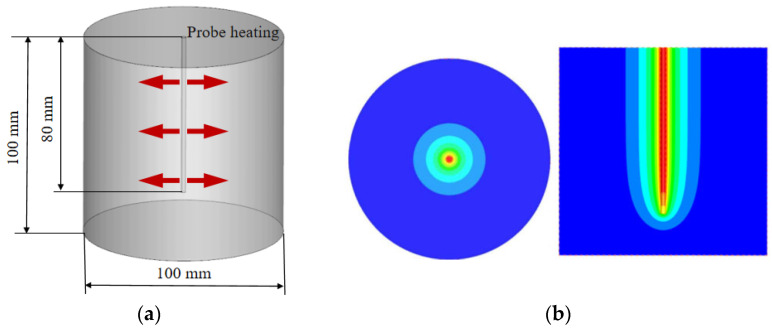
Schematic diagram of the heat transfer numerical model: (**a**) computational model; (**b**) calculated temperature field.

As shown in Figure 3, the numerical model was configured as a cylindrical soil column with the heating probe arranged in the center of the column. The diameter of the central probe was set at 2.50 mm. To simulate radial transient heat transfer in a semi-infinite space, the diameter of the soil column was set to 100 mm. The height of the soil column and the heating probe were 100 mm and 80 mm, respectively. Furthermore, based on the suggested heating power of the probe given by Ren et al. [30], the central probe was modeled as a Dirichlet boundary condition with a wall source term of 15 W/m. The outer profiles of the cylinder were defined as adiabatic boundary conditions. The physical properties of the soil column were chosen to be silty clay, the density was set to a constant value, and the heat capacity and thermal conductivity were modeled as segmented step functions with temperature. The specific values of soil physical properties were determined from Ref. [31].

A three-dimensional unsteady numerical model was developed, and all calculation cases were solved using the ANSYS 19.0 software package. The computational grid was generated using ICEM CFD 19.0 software, primarily with quadrilateral elements. An implicit algorithm was employed for solving the thermal conduction governing equations, and the diffusion term was discretized utilizing a second-order upwind difference scheme. The residual convergence criterion was set to 10^−5^. The total calculation time was 300 s, and the time step was defined as 1 s. Moreover, two probe heating modes were considered in the calculation: continuous heating (300 s) and discontinuous heating (heating stopped after 30 s).

### 2.3. Electromagnetic Simulation for Rationality Check of Probe Design Parameter

During the electromagnetic measurement process of the thermo-TDR sensor, the selection of probe spacing and diameter has a significant influence on the electromagnetic wave energy distribution around the probe. Unreasonable settings can easily lead to energy concentration and result in the skin effect [32], which, in turn, affects the accuracy of the dielectric parameter testing of three probes, ultimately obtaining inaccurate testing results of the unfrozen water content.

**Figure 4:**
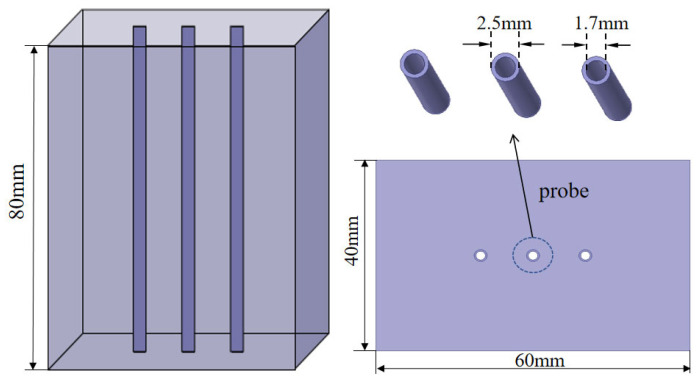
Diagram of the electromagnetic numerical model.

Therefore, a three-dimensional electromagnetic numerical model was developed to simulate the energy distribution characteristics around the probe with different spacing (also 6 mm, 8 mm, 10 mm, and 12 mm), checking the rationality of the given probe design parameters. As shown in Figure 4, the electromagnetic numerical model consisted of a cuboid soil block and three central layout heating probes. The geometry of the external soil region was defined as 40×60×80 mm, and the geometry of the three probes was consistent with the design parameters.

The governing equation of electromagnetic fields of TDR technology was ultimately expressed as Maxwell’s equations [33]:

(6) $$\nabla \cdot D\left(r,t\right)=p\left(r,t\right),$$

(7) $$D\left(r,t\right)=\varepsilon \left(r,t\right)\cdot E\left(r,t\right),$$

where *D*(*r*,*t*) represents electric displacement; *p*(*r*,*t*) denotes charge density; *ε* is the dielectric constant; *E*(*r*,*t*) signifies electric field strength.

Since the electric potential field around the TDR probe did not involve the physical charge, it was considered that *p*(*r*,*t*) = 0. Furthermore, it was known that *E*(*r*,*t*) was equal to the electric potential (∇Φ(*r*,*t*)). Therefore, the governing equation of the electric potential field around the TDR probe was simplified as:

(8) $$\nabla \cdot \left(\varepsilon \nabla \Phi \right)=0,$$

The boundary condition of the probe was set as an ideal conductor, while the boundary condition on the side surface of the cuboid soil block’s lateral surface was defined as a radiation boundary condition. The upper part of the block was defined as the excitation port, belonging to a Waveport type, and a fixed frequency was selected under different working conditions. Using the above simulation model, the potential field around the probe was calculated. Subsequently, the energy distribution characteristics and skin effect coefficients were analyzed to determine the reasonableness of the probe design parameters.

In this work, the area of the probe’s testing influence region under a certain cumulative proportion (*f*) was calculated by statistical method [34]. The detailed calculation procedures were as follows: (1) a large sampling region, which was sufficient to cover the entire probe testing influence range, was defined; (2) the whole sampling region discretized into adequate amount grids, while the integration value of *f* was 100% for all grids; (3) the weight function was used to calculate the *f*:

(9) $$f=\frac{100\cdot \sum_{w_{mi}}^{w}w_{i}A_{i}}{\iint w_{i}dA},$$

where *A_i_* is the area of certain statistical grid; *w_i_* is the weight function of the grid; *w_mi_* is the maximum weight function of all grids.

Based on the uniform distribution assumption of the dielectric constant in the TDR probe surrounding medium, the weight function of a certain grid location, *w*(*x*,*y*), was calculated by [35]:

(10) $$w(x,y)=\frac{{\left[\nabla \Phi (x,y)\right]}^{2}}{\left({\iint \left[\nabla \Phi \left(x,y\right)\right]}^{2}dxdy\right)},$$

where Φ(*x*,*y*) is the electric potential of a certain grid surrounding the probe.

The skin effect reflects the regional concentration of test-sensitive areas surrounding the TDR probe. In the numerical analysis, the skin effect coefficient, *Q,* was calculated by:

(11) $$Q=\frac{S_{f=50\%}}{S_{f=90\%}},$$

where *S_f_* = 90% is the area of the sampling region that has a 90% impact on the test results, and it is selected to represent the whole sampling region; *S_f_* = 50% is the area of the sampling region that has a 50% impact on the test results, representing the region that is extremely sensitive to the test results.

### 2.4. Validation Test for Proposed Thermo-TDR Sensor

**Figure 5:**
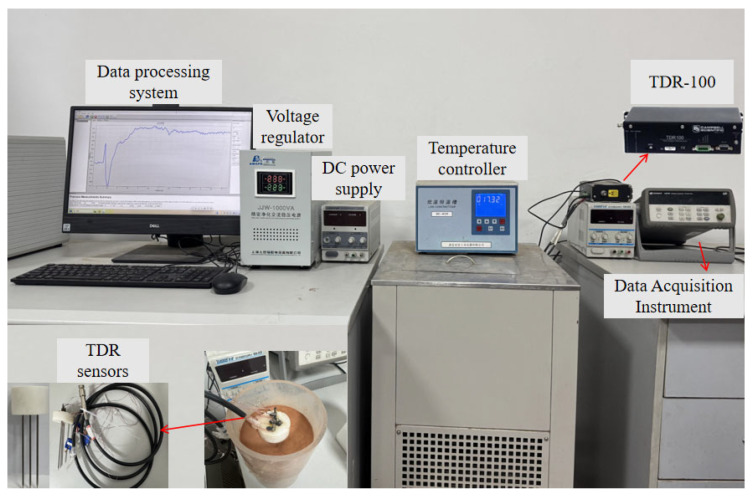
Validation experimental system for the proposed thermo-TDR sensor.

To validate the applicability of the proposed thermo-TDR sensor, a self-designed experimental system for simultaneous measurement of unfrozen water content and thermal conductivity of frozen soil was built. The experimental system was composed of a data acquisition instrument (Agilent 34970, Keysight Tech. Inc., Santa Rosa, CA, USA), a time domain reflectometer (TDR-100, Campbell Sci. Inc., Logan, UT, USA), a temperature-controlled bath (2200S, Yaxing Civil Instruments Co., Ltd., Xi’an, China), a data processing unit, a thermo-TDR sensor, and a DC power supply (RXN-302D, Zhaoxin Electronic Instrument Co., Shenzhen, China), as shown in Figure 5. The probe spacing and length of the manufactured thermo-TDR sensor adopted 10 mm and 80 mm, based on the analysis of thermal–magnetic simulations.

The sandy soil samples were collected from the Qinghai–Tibet Highway at the specific mileage of K2952 + 300 and K2917 + 871.5. The moisture content of test specimens was set as 6%, 8%, 10%, 12%, and 14%, and the dry density was chosen as 1.55 g/cm^3^. In order to approximate the semi-infinite linear heat source heating process, the diameter of the test soil sample was determined as 108 mm, and its height was set as 150 mm. The temperature measurement time of the thermo-TDR sensor was also set as 300 s, and a non-continuous 30 s heating strategy was adopted, employing a heating power of 15 W/m, as well. During the test, the soil sample was sealed with cling film and placed in the temperature-controlling bath to ensure that the soil sample was kept at the setting temperature. The testing temperature point started at −10 °C and gradually increased to the melted state. The detailed testing scheme is listed in Table 1.

It should be clarified that the probe parameters need to be calibrated before testing, mainly referring to the probe length and spacing. The probe length (*L*) calibration was performed in deionized water based on the empirical relationship between the dielectric constant of deionized water (*K_w_*) and temperature (*T*) provided in the literature [36]:

(12) $$K_{w}=84.740-0.40008T+9.398\times 10^{-4}T^{2}-1.410\times 10^{-6}T^{3},$$

Supposing *K_w_* is equal to *K_a_*, which is the dielectric constant of deionized water measured by the TDR sensor. The calibrated probe length can be calculated by:

(13) $$K_{a}={\left(\frac{c\Delta t}{2L}\right)}^{2},$$

where *c* is the light speed in vacuum conditions; Δ*t* is the time difference between the two reflection points of reflected waveforms.

The calibration of probe spacing was carried out utilizing agar solution at a concentration of 5 g/L [37]. The specific value of probe spacing (*r*) was calculated as follows [27]:

(14) $$\rho c=q/\left(e\pi r^{2}\Delta T_{m}\right)$$

where *q* is the heating power per unit length, choosing as 15 W/m; *ρ_c_* is the volumetric heat capacity of the agar solution, determining same as that of water, 4.18 MJ/(m^3^·K); *e* is natural logarithm; △*T_m_* is the maximum temperature rise at a distance of *r*.

Finally, the probe length and spacing of the manufactured thermo-TDR sensor were calibrated as 83.6 mm and 9.86 mm, respectively.

**Figure 6:**
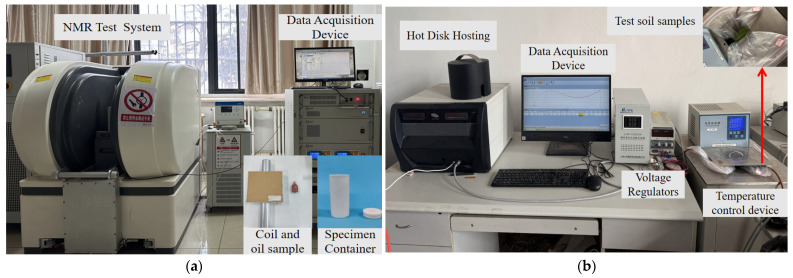
Comparative experimental system: (**a**) NMR test system. (**b**) Hot Disk test system.

Furthermore, to verify unfrozen water content and thermal conductivity measurement results of the thermo-TDR experimental system, comparative experiments were carried out using the nuclear magnetic resonance (NMR) method and the transient plane heat source method, as shown in Figure 6. The equipment used for the NMR test was a MesoMR low-temperature and high-pressure NMR analyzer (MAcroMr12, Suzhou Niumag Analytical Instruments Co., Ltd., Suzhou, China), which consisted of a permanent magnet, a radio frequency unit, and a data acquisition system. The test system of the transient planar heat source method was composed of a thermal conductivity analyzer (TPS 2200, Hot Disk Co., Ltd., Göteborg, Sweden), a temperature control device, and a data processing computer. Fine sandy soil with a moisture content of 10% and a dry density of 1.55 g/cm^3^ was selected for the comparison experiment.

## 3. Results and Discussion

### 3.1. Analysis of Thermal and Electromagnetic Simulation

#### 3.1.1. Optimization of Sensor Parameters

**Figure 7:**
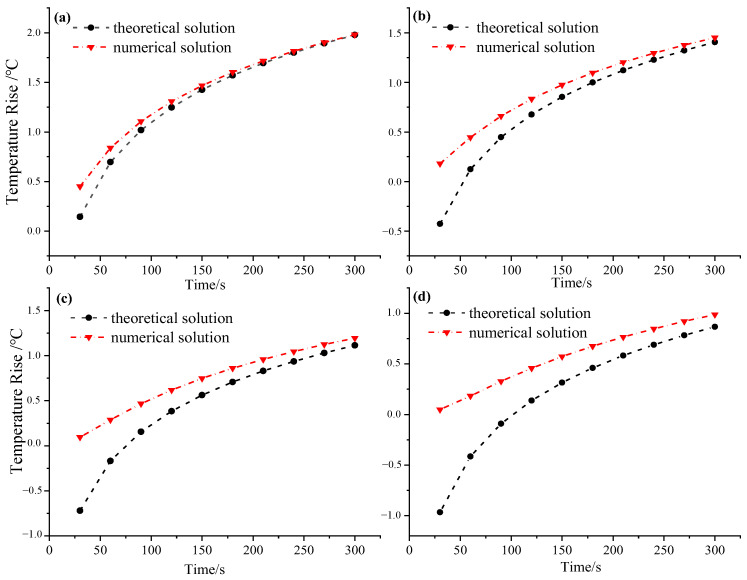
Comparison of theoretical and numerical temperature rise at varying probe spacings. (**a**) 6 mm. (**b**) 8 mm. (**c**) 10 mm. (**d**) 12 mm.

In order to optimize the design parameters of the thermo-TDR, the temperature rise of the soil during the heating of the probe and the deviation between the theoretical and numerical solutions of the thermal conductivity were analyzed. The theoretical and numerical temperature rise of the lateral probe, with the spacing of 6 mm, 8 mm, 10 mm, and 12 mm (distance to the heat source), were analyzed, and the results are shown in Figure 7.

It can be seen that the difference between the theoretical temperature rise and the numerical temperature rise decreases with increasing heating time at varying probe spacings. When the spacing is 8 mm, the difference between the theoretical and numerical temperature rise is 0.67 °C after 30 s of heating, and the difference is reduced to 0.05 °C after 300 s. Furthermore, with the increase in the probe spacing, the difference between the theoretical and numerical temperature rise becomes more significant; when the spacing is 6 mm, the difference between the theoretical and the numerical temperature rise after 300 s of heating is only 0.03 °C, and when the spacing is 12 mm, the difference between the theoretical and the numerical temperature rise increases to 0.12 °C under the same heating time. The main reason for this phenomenon is that as the probe spacing increases, the heat transfer path becomes longer, resulting in an increase in heat loss, which makes the difference between the theoretical calculation and the numerical simulation results more significant. Frozen soil testing requires a small increase in soil temperature and a large testing range. Therefore, it can be concluded that the continuous heating strategy of the thermal probe is not suitable for testing the physical parameters of frozen soil.

**Figure 8:**
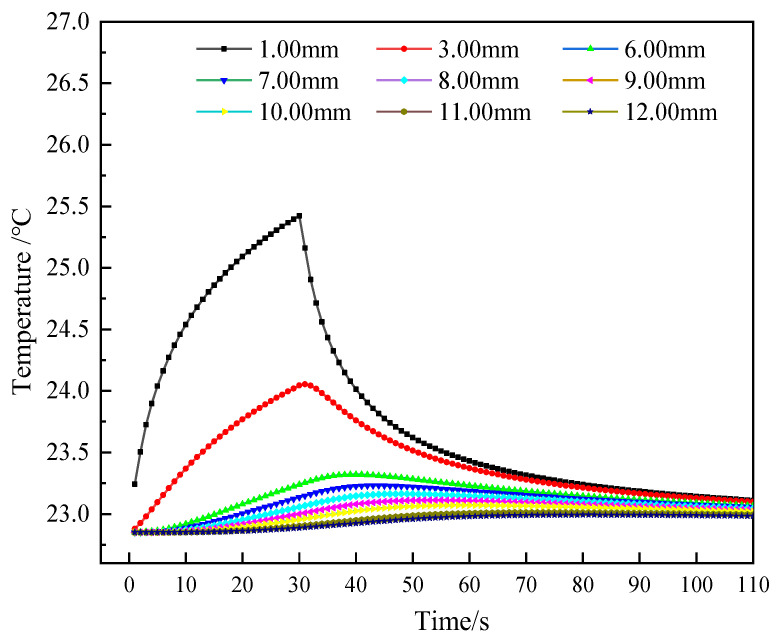
Temperature curves at varying probe spacings under a 30 s instantaneous heating scheme.

Thus, under a 30 s instantaneous heating scheme, the soil temperature curves at different locations were simulated. The calculation results are given in Figure 8. Moreover, the maximum temperature rise and their corresponding time, and the time to reach 90% maximum temperature rise (90% signal stabilization time) of different probe spacings were also collected, as listed in Table 2. Variations in probe spacing exert a notable influence on both thermal maxima magnitudes and their temporal occurrence patterns. As the probe spacing increases, the maximum temperature rise decreases, and the time required to reach the maximum temperature rise increases. When the spacing between the heating probe and the soil is 1 mm, the maximum temperature rise is 2.57 °C when the heating is stopped, and when the spacing is increased to 12 mm, the maximum temperature is 0.14 °C when the heating is stopped for 51 s. Through the analysis of the time required for the self-heating process to reach a 90% signal stability state under different probe spacings, it can be seen that with the increase in probe spacing, the 90% signal stabilization time shows a gradually increasing trend. When the probe spacing is 6 mm, 8 mm, 10 mm, and 12 mm, the 90% signal stabilization time is 33 s, 38 s, 45 s, and 59 s, respectively. This suggests that a larger probe spacing results in a longer time for the sensor to reach a steady state, but the overall difference is not significant. It can be seen that when the probe spacing is increased from 10 mm to 12 mm, the 90% signal stabilization time increases significantly. At the same time, from the perspective of the time required to reach the maximum temperature, the time increase at a spacing of 10 mm is relatively smaller compared to 12 mm.

**Figure 9:**
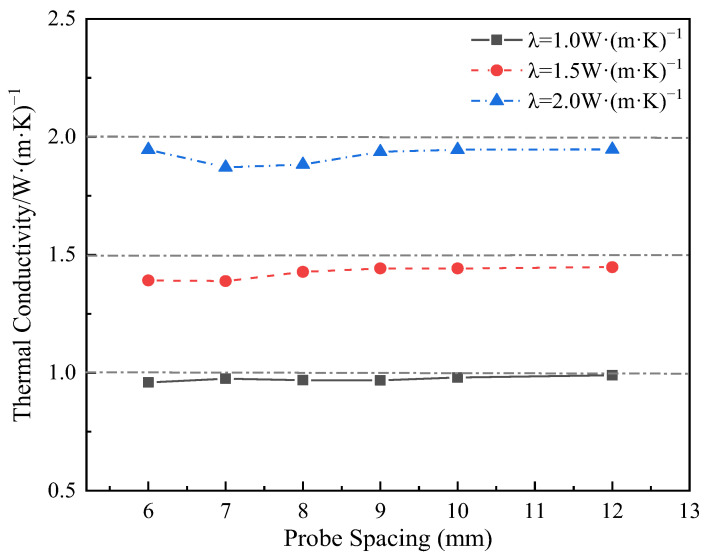
Comparison of theoretical solutions and preset values of thermal conductivity at varying probe spacings.

The thermal conductivity test results of the designed thermo-TDR sensor were obtained by theoretical calculation methods (as described in Section 2.1). Therefore, to verify the accuracy of the sensor’s test value of thermal conductivity, the numerical calculation was conducted with a preset thermal conductivity of 1, 1.5, and 2 W/(m·K), corresponding to the soil properties (gravelly clay, silty clay, and fully weathered mudstone). The results are shown in Figure 9. It can be seen that as the probe spacing increases, the deviation between the theoretical and set thermal conductivity gradually decreases. At 6 mm, 9 mm, 10 mm, and 12 mm probe spacing, the deviation rates were 4.53%, 3.40%, 2.94%, and 2.60%, respectively. Especially at a spacing of 12 mm, the deviation between the theoretical and set thermal conductivity is minimal. Considering that the temperature rise at 12 mm at room temperature is small (only 0.14 °C), and the temperature rise will be further reduced under negative temperature conditions, combined with the accuracy of the temperature sensor in the experiment (±0.1 °C), this temperature rise value may be difficult to measure accurately. Therefore, considering the accuracy of the thermal conductivity solution and the relationship between the temperature rise and the accuracy of the probe, it is reasonable to choose 10 mm as the probe spacing design.

#### 3.1.2. Energy Distribution Characteristic of Electromagnetic Field

Probe spacing and diameter have a significant effect on the energy distribution of electromagnetic waves around the probe. Unreasonable settings can easily lead to energy concentration around the probe, resulting in the skin effect, which in turn affects the accuracy of the dielectric parameter testing of the probe, ultimately leading to inaccurate moisture content test results.

**Figure 10:**
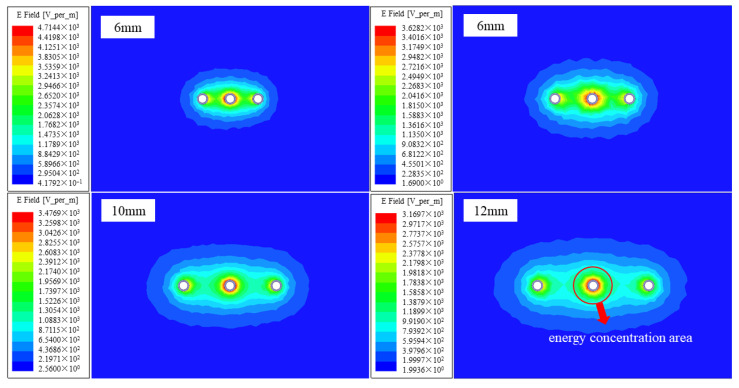
Distribution map of the plane electric field intensity for probes at varying probe spacings.

This section simulates the influence of different probe spacings on the energy distribution around the probe, given the known length and diameter of the probe. In the calculation process, attenuation, dispersion, and radiation loss during the propagation of electromagnetic waves were neglected, and the dielectric constant of the medium around the probe was assumed to be uniformly distributed. Therefore, the issue of the testing range around the probe can be simplified to a two-dimensional problem [33]. Figure 10 shows the calculated electric field intensity distribution on the probe plane at spacings of 6 mm, 8 mm, 10 mm, and 12 mm.

**Figure 11:**
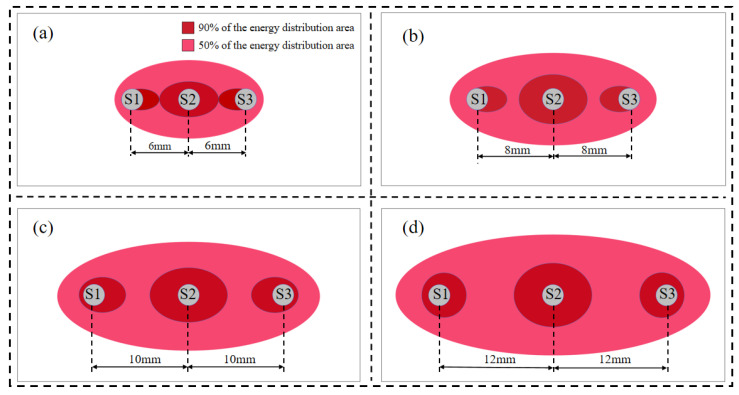
Distribution map of *S_f_* = 50% and *S_f_* = 90% regions at varying probe spacings. (**a**) 6 mm. (**b**) 8 mm. (**c**) 10 mm. (**d**) 12 mm.

It can be observed that the detection range of the probe is significantly expanded as the probe spacing gradually increases, and in all cases, the energy is concentrated at the intermediate probe. To better illustrate the concentration of energy at varying probe spacings, distribution maps of the *S_f_* = 50% and *S_f_* = 90% regions of the three-probe thermo-TDR probe are drawn, as shown in Figure 11. The specific calculation procedures are as follows: first, the data of each cell are derived from the electric field intensity distribution map, and the weight coefficient *w_i_* for each cell is computed; then, the coefficients *w_i_* are arranged in order of magnitude, and the testing area range parameter *f* is calculated.

**Figure 12:**
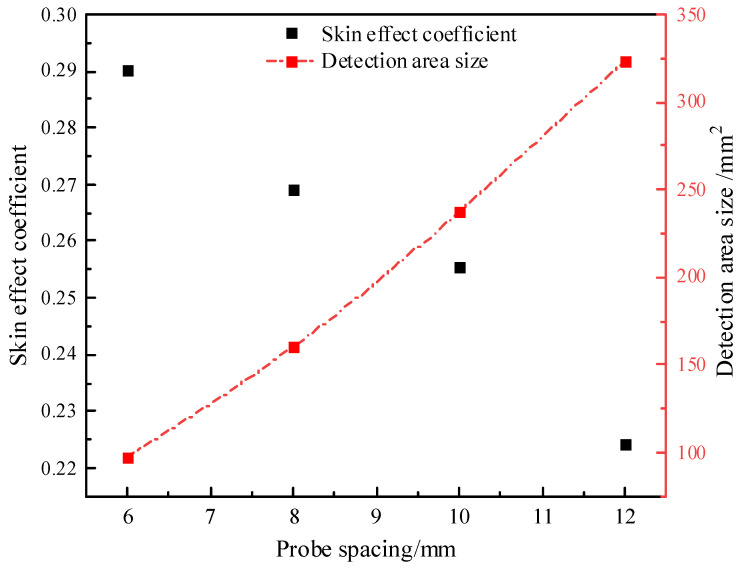
Variation of test medium range and skin effect coefficient with probe spacing.

Figure 11 illustrates the cross-sectional energy distribution of the electric field at varying probe spacings. When the spacing is between 8 mm and 10 mm, the spacing/diameter ratio falls within the range 0.2 < d/s < 0.4, which is consistent with the analysis results from Ferré et al. [34], verifying the reliability of the results in this study. It can also be observed that with the increase in spacing, the detection range becomes larger, and the *S_f_* = 50% energy increasingly concentrates around the probe. Under the same conditions, the areas of the detection range and skin effect values for different spacings are calculated, as shown in Figure 12. It can be seen that with the increase in spacing, the skin effect coefficient decreases, indicating that the areas with a greater influence on the test results become more concentrated, and the skin effect becomes more obvious, which is consistent with the expected trend. By employing a 10 mm probe spacing design, the test area is significantly increased to 237.6 mm^2^ and is 144.94% and 48.13% higher than that of the 6 mm and 8 mm spacing designs, respectively, and the reduction in the skin effect coefficient is relatively small; the skin effect coefficient of the thermo–TDR sensor is 25.54%, which satisfies the electromagnetic design requirements.

#### 3.1.3. Analysis of Significant Temperature Rise Scope

The physical properties of frozen soil are extremely sensitive to temperature changes [38]. In the testing process of the thermo-TDR sensor, the heat released by the center probe caused a significant temperature rise in the surrounding soil, resulting in test errors. In order to analyze the influence range of the heating process, we simulated the temperature rise at varying probe spacings in the environmental temperature range of −3~0 °C. The soil physical parameters utilized in the calculation were defined as the embankment fill soil in Wang et al. [31]. The statistical results are summarized in Table 3. It can be observed that the significant temperature rise position is close to the center probe, and its range is roughly 2~3 mm. As the environmental temperature increases, the maximum temperature rise of all positions gradually decreases. This is because not all the heat is used to raise the soil temperature during the heating process. A considerable account of the heat transforms to overcome the latent heat of the ice within the soil. Meanwhile, the environmental temperature is also a key influence factor. When the temperature approaches the phase transition temperature range, the impact of the ice/water phase transition becomes more significant, resulting in a reduction in the maximum temperature rise.

**Figure 13:**
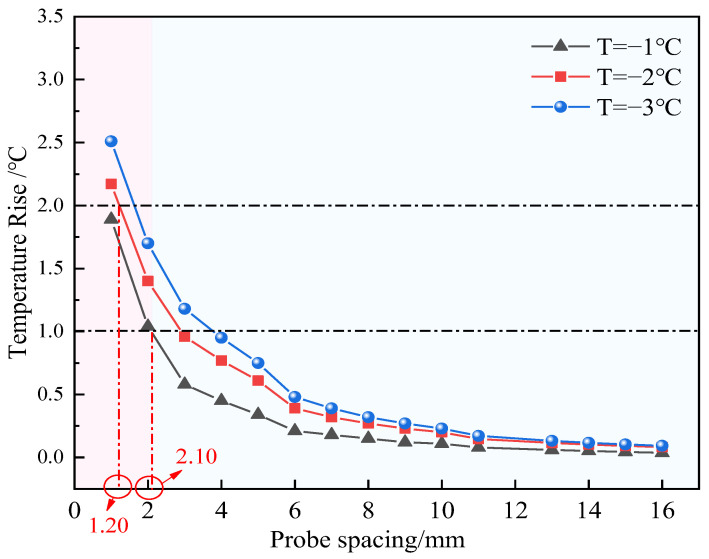
Maximum temperature rise at varying probe spacings and environmental temperatures.

The statistical results of the maximum temperature rise at varying probe spacings are shown in Figure 13. It can be seen that with the increase in spacing, the maximum temperature rise gradually converges under different conditions, indicating that the phase transformation caused by heating has less influence at a distance. When the environmental temperature is −1 °C, to ensure that the minimum temperature of the heating probe during the heat pulse measurement is below 0 °C and to prevent ice from melting, the maximum temperature rise should not exceed 1 °C. As shown in the figure, when the maximum temperature rise is 1 °C, the probe spacing is 2.10 mm. Similarly, when the environmental temperature is −2 °C and the maximum temperature rise is 2 °C, the probe spacing is 1.20 mm. When the environmental temperature is −3 °C, the maximum temperature rise does not exceed 3 °C. Thus, it can be concluded that under this heating mode, the probe spacing in the region with large temperature changes is approximately 0~2 mm, and the region with significant temperature rise is small, occupying a relatively minor volume proportion of the entire testing area. Therefore, taking all factors into consideration, it is more reasonable to set the probe spacing at 10 mm.

### 3.2. Analysis of Validation Experiments

#### 3.2.1. Validation Experiment of Unfrozen Water Content

**Figure 14:**
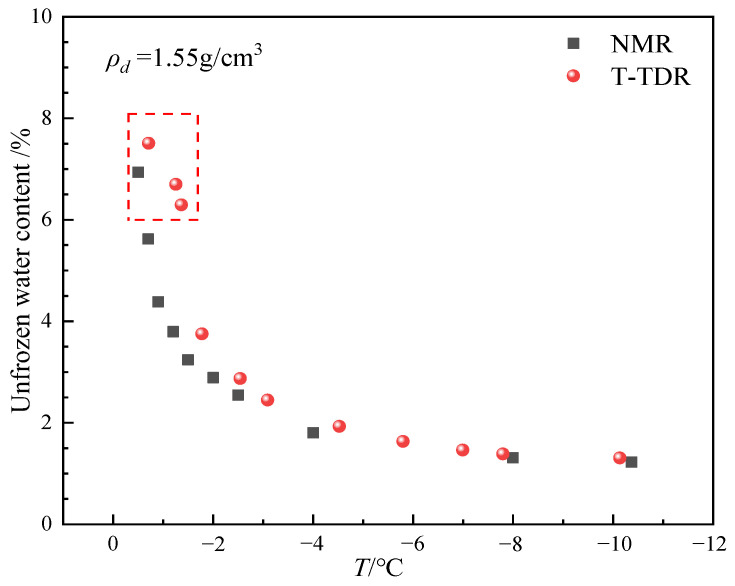
The variation of unfrozen water content measured by NMR and thermo-TDR methods at different temperatures.

Figure 14 shows the trend of unfrozen water content with temperature measured by the thermo-TDR and NMR methods for a sample with a moisture content of 10% and a dry density of 1.55 g/cm^3^. It can be seen that the unfrozen water content of the thermo-TDR test shows a similar trend change as the NMR method in a certain temperature range, which indicates that the thermo-TDR test method has good accuracy and can effectively capture the change of unfrozen water content in the permafrost. Furthermore, as the temperature decreases, the amount of unfrozen water will also gradually decrease. Within the temperature range of 0 °C to −2 °C, the unfrozen water content measured by TDR is generally higher than that measured by NMR. These discrepancies mainly arise from the ratio of ice-to-water in the frozen soil. The state of the ice/water mixture can influence the electromagnetic test data. The variation of unfrozen water in the −1 °C to −2 °C range is pronounced, and there are certain fluctuations in the temperature control device during the testing process, which objectively creates a temperature gradient in the soil and leads to dynamic fluctuations in the ice/water ratio, resulting in errors in the collected electromagnetic signals. In the temperature range of −3 °C to −10 °C, the unfrozen water content measured by both methods is relatively close, and at very low temperatures (close to −10 °C), the results of both methods converge, indicating that the effect of frozen water has become dominant. Although the thermo-TDR may have some measurement deviations in the frozen soil phase transition zone, it can still effectively reflect changes in unfrozen water content and has high accuracy, especially under low-temperature conditions.

**Figure 15:**
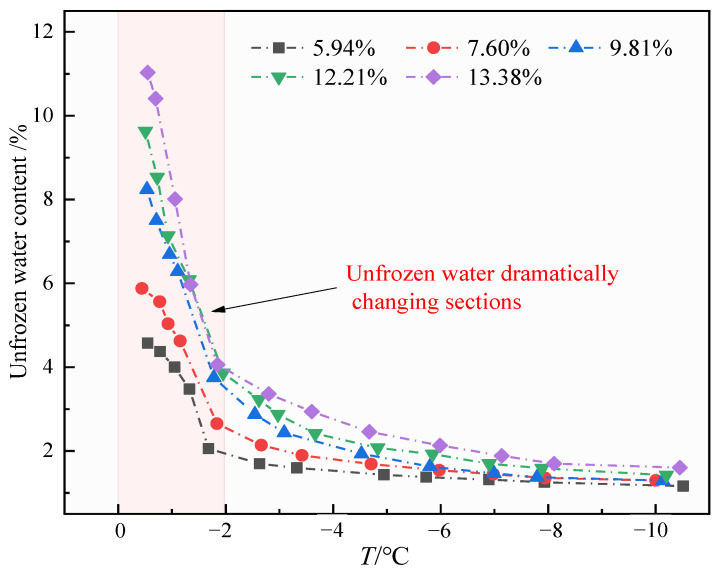
Change in unfrozen water content at different moisture contents and temperatures.

Statistical analysis of moisture content at different temperatures is shown in Figure 15. It can be seen that the unfrozen water content under different moisture content has the same trend with temperature, and the unfrozen water content gradually increases with the increase in temperature; the increase in range is also gradually accelerated, and the unfrozen water content changes drastically in the temperature range of −2 °C to 0 °C. Simultaneously, during the melting process, different initial moisture contents can lead to different unfrozen water contents at various temperatures. Soil samples with higher initial moisture content show correspondingly higher unfrozen water content. This is mainly because, when freezing, the samples with higher moisture content freeze more water. The ice exerts greater pressure on the pores, inhibiting the freezing of the remaining unfrozen water. Therefore, samples with higher moisture content generally have higher unfrozen water content. During the melting stage, the initially larger amount of unfrozen water absorbs heat, promoting further melting of the ice, which keeps the unfrozen water content higher in samples with higher initial moisture content.

After freezing the sample, due to capillary action and the adsorption effect on the surface of soil particles, not all liquid water in the soil will freeze. The unfrozen water maintains a dynamic equilibrium with temperature, and its expression is as follows [39]:

(15) $$W_{u}=a\theta^{-b},$$

where *w_u_* is the unfrozen water content; *θ* is the absolute temperature; *a* and *b* are constants related to the soil type.

**Figure 16:**
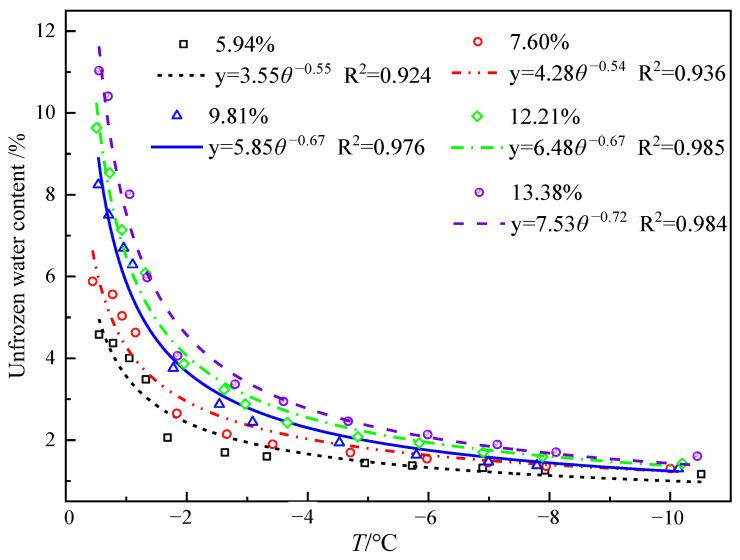
Fitting curve of unfrozen water content under varying moisture contents and temperatures.

Figure 16 illustrates the curves of unfrozen water content varying with temperature at different initial moisture contents. It can be seen that the relationship between the unfrozen water content and temperature at different moisture contents, measured by the thermo-TDR, shows a high degree of fitting, which verifies that the probe sensor has a certain accuracy in measuring the unfrozen water content. The unfrozen water content of fine sand soil shows an overall positive correlation with temperature. When the temperature is −10 °C, the unfrozen water content at different moisture contents is not equal to zero, indicating that there is some unfrozen liquid water in the sample at this time. This is because when the soil freezes, the pore water freezes completely, and the unfrozen water mainly consists of strongly bound water. As moisture decreases, the adsorption force of soil particles on strongly bound water increases, and the water film becomes thinner, making it more difficult for the strongly bound water to freeze. Therefore, even though the content of unfrozen water in the soil samples continues to decrease, there is still some water that has not completely frozen at extremely low temperatures.

#### 3.2.2. Validation Experiment of Thermal Conductivity

**Figure 17:**
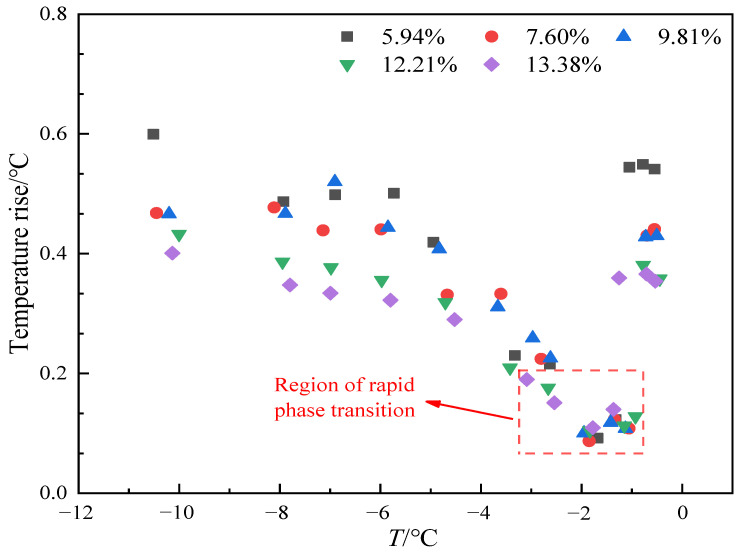
Variation curve of the maximum temperature rise of the outer probe at different moisture contents.

The variation in the maximum temperature rise of the outer probe at different temperature points under different moisture content conditions is shown in Figure 17. It can be seen that in the temperature range of −1 °C to −3 °C, the maximum temperature rise of the outer probe decreases significantly as the moisture content changes. Especially in the range from −1 °C to −2 °C, the temperature rise of the outer probe is not significant. This is mainly because the heat released by the heating probe is absorbed by the ice-melting process, which leads to a reduction in the heat transferred to the outer probe. Therefore, in the range between −1 °C and −2 °C, the phase transition between ice and water becomes more intense. Additionally, from the overall trend, the higher the moisture content in the soil, the smaller the maximum temperature rise of the outer probe at different temperatures. When the moisture content is 5.94%, the maximum temperature rise is 0.6 °C, while at a moisture content of 13.38%, the maximum temperature rise is 0.4 °C. This is due to the high specific heat capacity of water, which means that soil with a higher moisture content can absorb more heat to raise the water temperature, resulting in a smaller increase in soil temperature.

**Figure 18:**
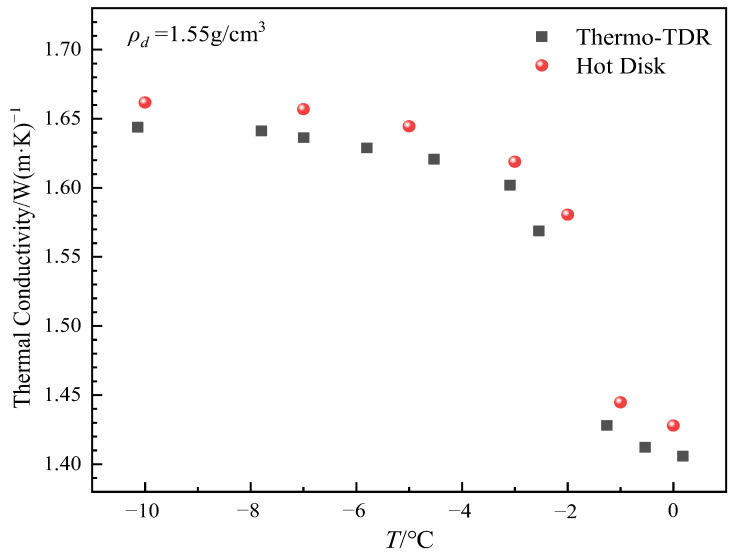
The variation of thermal conductivity measured by the transient plane heat source method and thermo-TDR method at different temperatures.

Based on the variation in the temperature rise curve of the outer probe, it is evident that the temperature rise is small in the −1 °C to −2 °C range, particularly between −1.5 °C and −2 °C, where the maximum rise is only approximately 0.1 °C. This can lead to large errors in the measurement of soil thermal conductivity. Therefore, the thermal pulse method is not suitable for determining soil thermal conductivity in this temperature range. The thermal conductivity of soil samples with a moisture content of 10% and a dry density of 1.55 g/cm^3^ measured by thermo-TDR and transient planar heat source methods is shown in Figure 18, and it can be seen that the trend of thermal conductivity measured by the two methods with temperature shows good consistency, which accurately reflects the trend of thermal conductivity increasing with temperature decrease. When the temperature decreases from 0 °C to −10 °C, the thermal conductivity measured by the transient planar heat source method increases from 1.423 W/m·K to 1.662 W/m·K, and the thermal conductivity measured by the thermo-TDR method increases from 1.412 W/m·K to 1.644 W/m·K. In general, the thermal conductivity measured by the transient planar heat source method is slightly higher than that measured by the thermo-TDR method, with a difference of about 0.018 W/m·K and an average relative deviation of 1.32%. Although there are certain differences in the measured thermal conductivity values, the range of these differences is not significant, and the error is generally within an acceptable range. It can be seen that the results measured by the thermo-TDR method have a certain degree of reliability.

**Figure 19:**
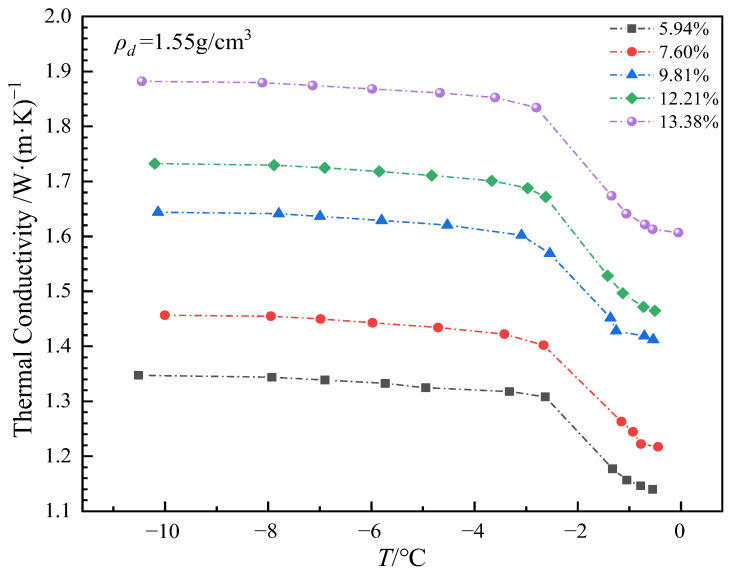
Variation of thermal conductivity with temperature at different moisture contents.

The thermal conductivity of soil samples with different moisture contents at different temperature points was solved and counted, as shown in Figure 19. It can be seen that the thermal conductivity of soil generally increases with the decrease in temperature, which is consistent with the change trend of unfrozen water content. During the phase transition zone, there is a significant increase in thermal conductivity. Below the phase transition zone, the rate of increase gradually slows, and the increase becomes smaller as the temperature decreases. This phenomenon is primarily caused by the decrease in temperature, which leads to the transformation of liquid water in the soil into solid ice. The volume of ice is about 1.1 times that of water. As the amount of ice increases, the soil pores are filled, and the contact area for heat flow increases, thus enhancing the soil’s thermal conductivity. Once the temperature falls below the phase transition zone, most of the water has been converted into ice, and the ice content stabilizes. The increase in thermal conductivity gradually decreases, and at extremely low temperatures, the thermal conductivity of ice and soil particles no longer undergoes significant changes. In general, with the increase in soil moisture content, the thermal conductivity of soil samples also increases. In the case of high moisture content, the overall increase in thermal conductivity is more significant, while in the case of low moisture content, the increase is slowed down. In low moisture content soils, although the moisture content is small, the decrease in temperature can promote the freezing of limited water, which can still improve the thermal conductivity to a certain extent. For high moisture content soil, a large amount of water freezes to form a more continuous ice network structure, and the heat conduction path increases and is more efficient, resulting in a significant increase in thermal conductivity, and under the condition of high moisture content, the freezing of water leads to frost heave phenomenon, and due to the restraint effect of the mold, the soil particles are further compressed, so that the thermal conductivity is further increased. In contrast, the low moisture content soil has a relatively low thermal conductivity and a small increase due to the low amount of ice filling in the pores of the soil, which has a limited effect on soil particle arrangement and thermal contact.

## 4. Conclusions

In this work, a thermo-TDR sensor design was proposed for measuring the unfrozen water content and thermal conductivity of frozen soil. The heat transfer and electromagnetic numerical models were developed to analyze the proper probe spacing and energy distribution characteristics of the thermo-TDR sensor. Finally, the validation experimental system of the thermo-TDR sensor was built, and the test results were compared with those of the NMR and transient planar heat source methods to confirm the usability and accuracy of the designed sensor. The following conclusions are drawn.

(1) The transient heating scheme has minor thermal disturbance to the surrounding soil, which is more reasonable for the measurement of the hydrothermal parameters of frozen soil. The deviation rate between the theoretical calculated thermal conductivity and the numerical preset value is only 2.94%, which confirms the sensor’s accuracy in thermal physics testing.
(2) By employing a 10 mm probe spacing design, the test area is significantly increased to 237.6 mm^2^ and is 144.94% and 48.13% higher than that of the 6 mm and 8 mm spacing designs, respectively. With 10 mm probe spacing, the skin effect coefficient of the thermo-TDR sensor is 25.54%, which satisfies the electromagnetic design requirements.
(3) The experimental results of the designed thermo-TDR sensor have a good consistency with those obtained from NMR and transient plane source methods, validating that the thermo-TDR sensor is capable of measuring the unfrozen water and thermal conductivity simultaneously. It is also noted that the hydrothermal parameters of frozen soil exhibit a drastic variation trend in the temperature range of −2~0 °C.
(4) Testing errors of the thermo-TDR sensor will significantly increase in the near-phase transition range, especially in the range of −2~−1 °C. Therefore, determination and correction studies on the test errors induced by drastic changes in soil’s physical properties during the probe’s heating process need to be conducted in the future.

The designed thermo-TDR sensor in this work successfully achieves synchronous measurements of unfrozen water content and thermal conductivity of sandy soil. Compared to the traditional separate measurement methods, the thermo-TDR sensor exhibits significant advantages in terms of experimental operation simplification and data acquisition efficiency, which provides an innovative technical approach for geothermal physical property testing and in situ permafrost monitoring in cold regions. In the future, we will further explore the test methodology of the proposed thermo-TDR sensor in practical engineering application scenarios, including sensor deployment methods under different soil conditions, improving corrosion resistance and stability, and analyzing the influence of ground temperature gradient and humidity fluctuation on test results.

## Figures and Tables

**Table 1:** Testing scheme design for the thermo-TDR sensor.

Specimen Number	Moisture Content/%	Dry Density/g·cm^−3^	Temperature Point/°C
A1	6	1.55	0, −0.5, −0.9, −1.2, −1.7, −2.7, −3.5, −4.7, −5.7, −6.7, −7.7, −10
A2	8	1.55	
A3	10	1.55	
A4	12	1.55	
A5	14	1.55	

**Table 2:** Maximum temperature rise and their corresponding times, and 90% signal stabilization time at varying probe spacings.

Spacing (mm)	1.00	3.00	5.00	6.00	7.00	8.00	9.00	10.00	12.00
Maximum Temperature Rise (°C)	2.57	1.20	0.75	0.47	0.38	0.31	0.26	0.22	0.14
Time to Reach Maximum Temperature (s)	30	31	34	40	44	48	53	59	81
90% Signal Stabilization Time	22	26	29	33	35	38	41	45	59

**Table 3:** Maximum temperature rise at varying probe spacings.

Spacing (mm)	1	2	3	4	5	6	7	8	9	10
−1 °C	1.89	1.04	0.58	0.45	0.34	0.21	0.18	0.15	0.12	0.11
−2 °C	2.17	1.40	0.96	0.77	0.61	0.39	0.32	0.27	0.23	0.20
−3 °C	2.51	1.70	1.18	0.95	0.75	0.48	0.39	0.32	0.27	0.23

## Data Availability

The data presented in this study are available on request from the corresponding author.
